# In Vitro Analysis of CsA-Induced Hepatotoxicity in HepG2 Cell Line: Oxidative Stress and α2 and β1 Integrin Subunits Expression

**DOI:** 10.5812/hepatmon.11447

**Published:** 2013-08-13

**Authors:** Zohreh Mostafavi-Pour, Fatemeh Khademi, Fatemeh Zal, Ahmad Reza Sardarian, Fatemeh Amini

**Affiliations:** 1 Biochemistry Department, Medical School, Shiraz University of Medicinal Sciences, Shiraz, IR Iran; 2 Recombinant Protein Laboratory, School of Advanced Medicinal Sciences and Technologies, Shiraz University of Medicinal Sciences, Shiraz, IR Iran; 3 Reproductive Biology Department, School of Advanced Medical Sciences and Technologies, Shiraz University of Medical Sciences, Shiraz, IR Iran; 4 Student Research Committee, Department of Orthodontics, Dental School, Shiraz University of Medical Sciences, Shiraz, IR Iran

**Keywords:** Cyclosporine, Antioxidant, Enzymes, Integrin alpha2beta1

## Abstract

**Background:**

Cyclosporine A (CsA)-induced hepatotoxicity could be due to a reduction in α2β1 integrin expression that may either be from the direct effect of CsA itself or from reactive oxygen species (ROS) overproduction.

**Objectives:**

In this study we aimed to identify the cellular mechanisms underlying CsA-induced hepatic injury by investigating the activation patterns of the antioxidant enzymes, using HepG2 as an *in vitro* model.

**Materials and Methods:**

HepG2 cells were cultured with different concentrations of CsA (0, 0.1, 1, 10 μg/ml) for 72 h. Effect of CsA on, 1) cellular integrity, 2) glutathione reductase (GR) and glutathione peroxidase (GPx) activity, 3) cellular levels of glutathione (GSH), 4) intracellular ROS, 5) ALT and AST activities, 6) urea production and 7) α2β1 integrin expression were assayed.

**Results:**

CsA treatment demonstrated a dose dependent increase in intracellular levels of ROS, GPx activity and decrease in GSH levels (P<0.05). GR activity was mildly attenuated in 1 and 10 µg/ml concentrations of CsA. Alanine aminotranferase (ALT) and aspartate aminotransferase (AST) levels increased in CsA treated cells, while urea synthesis was significantly decreased following treatment with higher concentrations of CsA (P<0.05). Significant down-regulation of β1integrin expression was observed in 1 and 10 µg/ml CsA treated cells while α2 integrin mRNA was significantly down-regulated in all CsA treated cells.

**Conclusions:**

The observed reduction of α2β1 integrin expression following CsA treatment could be proposed as a possible pathway of CsA-induced hepatotoxicity. Further studies are required to elucidate whether this attenuated expression is due to the direct effect of CsA or caused by overproduction of ROS.

## 1. Background

Cyclosporine A (CsA) an 11-amino acid cyclic polypeptide isolated from the fungus *Topocladium inflatum*, is a powerful immunosuppressive drug that is widely used to prevent rejection of organ transplants and to manage various autoimmune conditions such as rheumatoid arthritis and nephritic syndrome (1, 2). However therapeutic benefits of this drug are limited by several side effects including renal, hepatic, cardiac, alimentary, and neural toxicity (3-5). Hepatotoxicity has been reported in both transplant and non-transplant conditions (autoimmune disorders).

Although the precise mechanisms by which CsA causes hepatotoxicity are not completely clarified, several investigators have suggested that CsA induced hepatotoxicity may be the sequel of reactive oxygen species (ROS) production, oxidative stress and depletion of hepatic antioxidant system (6-8). It has been reported that exogenous ROS significantly impairs adhesion and spreading of mesenchymal stem cells (MSC) and scavenging ROS successfully restores adhesion and spreading of these cells (9).

Integrins are the mediators of the molecular dialogue between a cell and its extracellular matrix environment (10). Integrin α2β1 is known as the principal collagen receptor and is crucial for the regulation of collagen metabolism. It has been reported that in CsA-induced gingival overgrowth, fibroblasts had lower expression of α2 integrin than the control but the mRNA expression of β1 integrin was not affected (11). However, to date, studies clarifying alterations in the expression of integrins in CsA-induced hepatotoxicity have not been conducted.

## 2. Objectives

In this study we analyzed the hepatotoxic effects of CsA on HepG2 cells focusing on changes in the activation pattern of antioxidant enzymes glutathione peroxidase (GPx) and glutathione reductase (GR), glutathione (GSH) levels, intracellular ROS production, supernatant alanine aminotranferase (ALT) and aspartate aminotransferase (AST) levels and α2β1 integrin expression.

## 3. Materials and Methods

### 3.1. Materials

Glutathione reductase (GR), tert-butyl hydroperoxide (t-BuOOH), bovine serum albumin (BSA), Triton X-100, di-methyl sulfoxide (DMSO) were purchased from Sigma Chemical Co (Poole, Dorset, UK); Na2-NADPH, di-sodium hydrogen phosphate (anhydrous) were obtained from Fluka Chemical Co (Buchs, Switzerland). Sodium azide, sodium chloride, magnesium chloride and EDTA were obtained from Merck (Darmstadt, Germany). Trypsin was from BDH-England. Potassium chloride, potassium dihydrogen orthophosphate were from Fluka-England. RPMI, FBS (fetal bovine serum), Penicillin and streptomycin were obtained from Gibco-BRL (Paisley, UK) and CsA was provided by Neoral, Novartis Pharma (Basel, Switzerland). Tripure Isolation Reagent was from Roche Applied Sciences (Indianapolis, IN). cDNA Synthesis Kit was purchased from Fermentas, EU. SYBR green DNA PCR Master Mix was from AB Company (Foster City, CA USA).

### 3.2. Cell Culture and Treatment

The human hepatocellular cell line (HepG2) was obtained from NCBI (National Cell Bank of Iran, Pasteur Institute, Tehran). HepG2 cells were cultured in RPMI-1640 medium supplemented with 10% FBS, penicillin (100 IU/ml) and streptomycin (100 μg/ml) at 37 °C in 5% CO2. The cells were seeded at a density of 5×10^5^ cell in a flask (25 cm^2^) and then incubated with different concentrations of CsA (0, 0.1, 1, 10 μg/ml) for 72 h (12).

### 3.3. MTT Assay

A cell viability assay was carried out using 3-(4,5-dimethyl-thiazol-2-yl)-2,5-diphenyltetrazolium bromide (MTT) as described by Mosmann (13), with minor modifications. MTT assay is a test to monitor mitochondrial respiration. It is a colometric assay that relies on the conversion of yellow tetrazolium bromide (MTT) to purple formazanderivative by mitochondrial succinate dehydrogenase in viable cells. Briefly, HepG2 cells (1x104cells/well in 96-well plate) were first incubated with 0.1, 1 and 10 μg/ml CsA for 72 h at 37ºC. The cells were then incubated in serum-free medium to which MTT (0.5 mg/mL, 10 μl) was added. Following 3.5 h of incubation, 100 μl of DMSO was added to dissolve the formazan crystals and the absorbance was determined in an ELISA reader at 570/650 nm. The number of metabolically competent cells was determined as the ratio (expressed as a percentage) of absorbance of treated cells to untreated cells that served as a control. The experiment was repeated five times.

### 3.4. Determination of Intracellular Generation of ROS

DCFH-DA fluorescent probes were used to measure the intracellular generation of hydroperoxide (H2O2) and superoxide anions (O2˙ˉ), respectively (14). This probe is a stable nonpolar compound that readily diffuses into cells. Once inside the cells, the acetate groups of DCFH-DA are cleaved from the molecule by intracellular esterases to yield DCFH, which is trapped within the cells. Intracellular H2O2 or low-molecular weight peroxides, oxidize DCFH to DCF, a highly fluorescent compound. Thus, fluorescence intensity is proportional to the amount of peroxides produced by the cells. Briefly, HepG2 cells were seeded at a density of 1x10^4^ cells/well in a 96-well plate. Following the cell treatments, the media of each well was removed and then 100 μl of 10 μM DCFH-DA was added to each well, then incubated for 20 min at 37 ºC in a humidified 5% CO2 atmosphere. After incubation, the extracellular DCFH-DA was replaced with 200 μl of PBSˉ and fluorescence was monitored using a Fluorimeter at 480 and 530 nm for excitation and emission.

### 3.5. Determination of Glutathione Peroxidase (GPx) Acivity

The procedures described by Fecondo and Augustey (15), which monitor continuous regeneration of reduced glutathione from oxidized glutathione (G-S-S-G) in the presence of glutathione reductase (Sigma Chemical Company, USA) and disodium (Na2) salt of reduced nicotinamide adenine dinucleotide phosphate (NADPH; Fluka Chemical Company, Switzerland) was used for the determination of GPx activity with minor modifications (16). The enzyme activity in the clear supernatant of HepG2 cell lysate was expressed as μmol of NADPH oxidized/min/mg of cell protein using a molar extinction coefficient of 6.22×106 M-1cm-1 for NADPH. One unit of GPx is defined as mU/mg of cell protein.

### 3.6. Determination of Glutathione Reductase (GR) Activity

The activity of GR was assayed using the method described by Racker with minor modifications (17). The GR assay was performed in a cuvette in a total volume of 1 ml that contained 60 μM buffer, 5 mM EDTA (pH 8.0), 0.033 M GSSG, 2 mM NADPH, and a sample in a final volume of 1 ml. The decrease in absorbance, which reflects the oxidation of NADPH during reduction of GSSG by GR present in the sample, was monitored spectrophotometrically at 340 nm for 3 min. Results were based on a molar extinction coefficient for NADPH of 6.22×106M-1cm-1. One unit of GR is defined as mU/mg cell protein.

### 3.7. Determination of Reduced Glutathione

The assay of GSH with DTNB was performed followed by a standard Ellman’s method (18). Standard curves were made from 1mM solutions of GSH. Clear supernatant of cell lysate was analysed for GSH level. 2.3 ml of potassium phosphate buffer (0.2 M, PH 7.6) was added to 0.2 ml of cell lysate supernatant and then 0.5 ml of DTNB (0.001 M) was added to the solution. The absorbance of the products was observed after 5 min at 412 nm.

### 3.8. Determination of ALT, AST and Urea Production

Following the 72 h treatment, cell culture media from each of the 25 cm2 culture flasks was collected, centrifuged at 3000×g for 10 min and stored at -70°C until assay. Urea level in the cell culture supernatant was determined by the urea nitrogen diagnostic kit (Parsazmoon) that utilizes an urease/Berthelot, quantitative, colorimetric method (19). ALT and AST activities were measured using a kit (ZistChimie, Tehran, Iran) based on the colorimetric method of Reitman and Frankel (20).

### 3.9. Semi Quantitative RT-PCR Analysis of α2 and β1 Integrin Subunits Expression

Total RNA was extracted by Tripure isolation reagent (Roche) and used for cDNA synthesis. Reverse transcription was performed using revert aid first strand cDNA synthesis kit (Fermentase) following the manufacturer's instruction. After an initial denaturation for 5 min at 95 ºC, the samples were amplified for 35 cycles; annealing at 45 ºC for 45 sec, elongation at 72ºC for 1 min, and denaturation at 95 ºC for 45 sec. The duration of the final elongation reaction was increased to 10 min at 72 ºC to permit completion of reaction products. The PCR products were separated on a 1.5% (w/v) agarose gel and visualized by Gel-Red staining. Primer sequences were β actin (Forward: 5΄-GCTGTGCTACGTCGCCCTG-3΄, Reverse: 5΄-GGAGGAGCTGGAAGCAGCC-3΄), β1 integrin (Forward: 5΄-GAAGGGTTGCCCTCCAGA-3΄, Reverse: 5΄-GCTTGAGCTTCTCTGCTGTT-3΄) and α2 integrin (Forward: 5 ΄-GGAACGGGACTTTCGCAT-3΄, Reverse: 5΄-GGTACTTCGGCTTTCTCATCA-3΄).

### 3.10. Real Time RT-PCR Analysis of α2 and β1 Integrin Subunits Expression

Our real time PCR was performed on an Applied Biosystem (AB) and SYBER Green І DNA-binding dye for real time PCR amplification detection was used. Briefly, PCR reactions were performed with the following conditions:

After an initial denaturation for 10 min at 95 ºC, the samples were amplified for 40 cycles; annealing at 60 ºC for 30 sec, elongation at 60 ºC for 30 sec, and denaturation at 95 ºC for 15 sec in the ABI 7500 system (AB, USA).

### 3.11. Statistical Analysis

The effect of the varying concentrations of CsA was assessed by various study parameters using a repeated measures experimental design. All data were analyzed by Kruskal-Wallis and Mann-Whitney tests for group comparison and expressed as mean ± SEM. P<0.05 was considered as significant. SPSS 15 software was used for data analysis.

## 4. Results

### 4.1. Effect of CsA Treatment on Cell Survival in HepG2 Cells

MTT test was used as an indicator of cytotoxicity induced by CsA in cultured HepG2 cells. The cells were exposed to increasing concentrations of CsA from 0-10 µg/ml for 72 h and maximal ethanol concentration used to solubilize and dilute CsA as vehicle. Our results showed that treatment with 0.1 and 1 µg/ml of CsA had no effect on cell viability while 10 µg/ml of CsA induced 35% cell damage (Data not shown).

### 4.2. Effect of CsA on ROS Production

**Figure 1.**
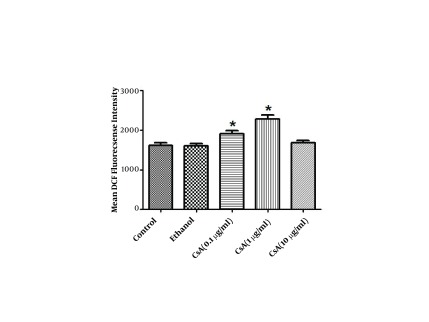
The Effect of CsA on ROS Production in HepG2 Cells

The intracellular formation of ROS was determined by using the DCFH-DA fluorescence method. According to our data, HepG2 cells treated with 0.1 and 1 µg/ml concentrations of CsA significantly increased ROS production by 18% and 40% respectively while the amount of produced ROS did not change following treatment with 10µg/ml CsA (P < 0.05) (Figure 1). 

### 4.3. The Effect of CsA on GPx Activity

**Figure 2.**
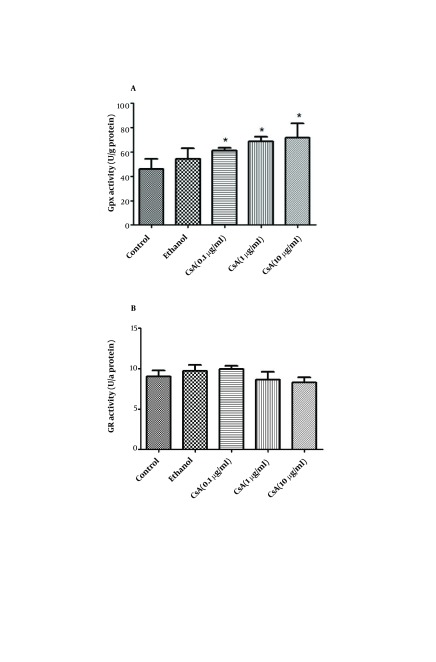
The effect of CsA on the activity of (A) glutathione peroxidase and (B) glutathione reductase in HepG2 cultured cells. HepG2 cells were incubated with 0, 0.1, 1 and 10 µg/ml of CsA and maximal ethanol concentration was used to solubilize and dilute CsA as vehicle, for 72 h. Data are presented as mean ± SEM. Sample size (n = 4), P<0.05 for significant change as compared to the control (no treatment).

As demonstrated by Figure 2 A, GPx activity was increased by 32%, 50% and 56% in 0.1, 1 and 10 µg/ml of CsA concentrations in comparison with the control (no treatment) and this increase was significant in 0.1 and 1 µg/ml (P < 0.05).

### 4.4. The Effect of CsA on GR Activity

Treatment of HepG2 cultured cells with different concentrations of CsA, resulted in a diminished activity of GR in 1 and 10 µg/ml of CsA concentrations but this reduction was not significant. As indicated in Figure 2 B, 0.1 µg/ml of CsA, had a mild increasing effect on GR activity.

### 4.5. The Effect of CsA on GSH

**Figure 3.**
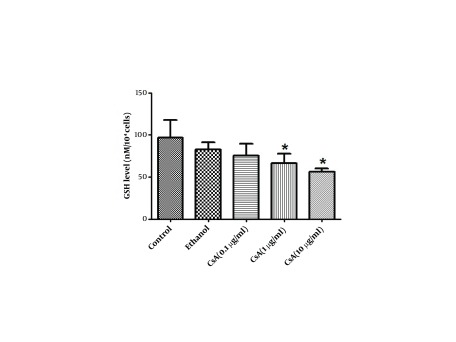
The Effect of CsA on GSH Level

As shown in Figure 3, the GSH level in HepG2 cell lysate after 72 h of CsA treatment was significantly reduced by 32% in 1 µg/ml and 42% in 10 µg/ml of CsA when compared to the control group (P < 0.05). 

### 4.6. The Effect of CsA on Mature Hepatocyte Markers

**Figure 4.**
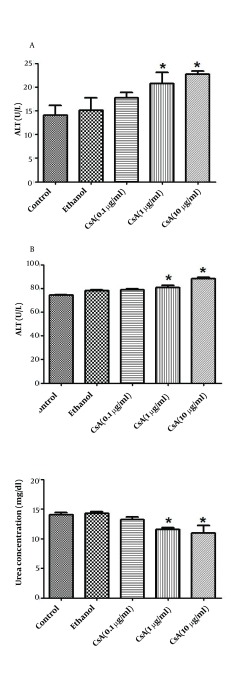
ALT, AST and Urea Concentration in Cultured Medium Was Measured in Untreated and CsA Treated HepG2 Cells Confluent cells in 25 cm2 flasks at a density of 5 × 105 cells were incubated with increasing doses of CsA from 0 to 10 µg/ml for 72 h with maximal ethanol concentration used as a vehicle. Cell culture media was centrifuged at 3000 × g for 10 min to remove debris. Supernatants were collected and stored at -20 ºC until used. A) ALT; B) AST were measured based on the colorimetric method; C) Urea measurement was done by urease/Berthelot, quantitative, colorimetric method. Data are expressed as mean ± SEM. Sample size (n = 3), P < 0.05 for significant change as compared to control (no treatment).

Cell damage associated with CsA was assayed by the measurement of ALT, AST and urea production. According to the results, ALT and AST production was increased significantly in 1 and 10 µg/ml concentrations of CsA as compared to the control (no treatment) (P < 0.05) (Figure 4 A and B). As shown in Figure 4 C, CsA treatment significantly diminished urea production in 1 and 10 µg/ml concentrations of CsA by 18% and 22% respectively as compared to the control (P < 0.05).

### 4.7. Semi Quantitative RT-PCR Analysis of α2 and β1 Integrin Subunits Expression

**Figure 5.**
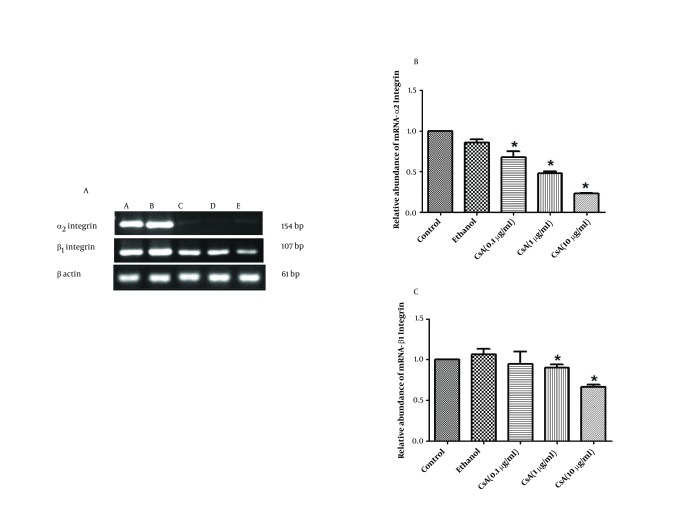
A) Gel-Red stained-agarose gel electrophoresis of RT-PCR products of five experimental groups in HepG2 cells. 4 µg of RNA was reverse transcribed and cDNA was amplified for 35 cycles. A- Normal HepG2 cells received no treatment (control), B-HepG2 cells received absolute ethanol as vehicle, C-HepG2 cells received 0.1 µg/ml CsA, D-HepG2 cells received 1 µg/ml CsA, and E- HepG2 cells received 10 µg/ml CsA; B) RNA levels of α2integrin and C) RNA levels of β1integrin subunits in five experimental groups. The RNA level of given integrin subunits were determined by real-time RT-PCR. Data are presented as mean ± SEM. Sample size (n = 3), P < 0.05 for significant change as compared to the control (no treatment).

RT-PCR results revealed that, although β1 integrin subunit expression is diminished in CsA treated groups compared to the control, the effect of CsA on the inhibition of β1 integrin subunit expression is much less than its inhibitory effect on α2 integrin subunit expression (Figure 5 A).

### 4.8. Real Time RT-PCR Analysis of α2 and β1 Integrin Subunits Expression

To determine whether CsA induces modifications in the pattern of α2 and β1 integrin subunits expression, we compared the level of RNA encoding for these two genes in five experimental groups. Alpha2 integrin mRNA quantification, revealed a significant down-regulation in 0.1, 1 and 10 µg/ml CsA treated groups, by 32 %, 52 % and 77 % respectively as compared to the control (Figure 5 B). Also quantification of β1integrin mRNA revealed a significant down-regulation in 1 and 10 µg/ml concentrations by 10 % and 34 % as compared to the control (Figure 5 C).

## 5. Discussion

The aim of the present study was to observe the effects of CsA treatment on HepG2 cells as a means to better understand the events underlying CsA-induced hepatotoxicity. We found that CsA treatment diminished the levels of reduced GSH and α2β1 integrin expression while increasing hepatic injury markers (ALT and AST).

The effect of CsA treatment on cell viability was determined and similar to the findings of Aker et al. on MDCK cells; we found that treatment with 10µg/ml of CsA induced 35% cell damage suggesting CsA specific toxicity to HepG2 cells with this dose.

In the present study, CsA treatment produced a significant elevation in intracellular generation of ROS and changes in glutathione homeostasis suggesting a role for oxidative stress and ROS production in CsA cytotoxicity. It should be mentioned that the reduction in ROS production observed in treatment with 10µg/ml CsA is attributed to the high level of cell death occurring at this concentration (35%). Indeed some authors have shown that cellular integrity is affected by oxidative stress when the production of active oxidants exceeds the capabilities of the antioxidant defense mechanisms ( 21 , 22 ). When ROS begin to accumulate, hepatic cells exhibit a defensive mechanism by using various antioxidant enzymes. The main peroxide detoxifying system involves GSH and the enzymes involved in GSH production (GPx and GR) ( 23 ). As shown in Figure 2, following HepG2 cell treatment with different concentrations of CsA, GPx activity increased but the activity of GR decreased. Although some observations indicated that CsA treatment caused decreases in GPx activity ( 16 , 24 ), findings of Puerto et al are compatible with our findings. They reported an enhancement of GPx activity and reduction of GR activity. In agreement with our findings, Bermejo-Bescos et al. ( 25 ) also demonstrated that oxidative stress decreased GR activity ( 25 ). 

Our results suggest that oxidative stress induced by CsA may occur because of the non-coordinated activity of primary antioxidant defense enzymes. GPx in concert with GR function to protect the cells from damage was inflicted by ROS. GPx detoxifies peroxides resulting in the production of GSH as an end product. Glutathione is a small protein composed of three amino acids, (cysteine, glutamic acid and glycine). It is an important antioxidant and plays a very important role in the defense mechanism of tissues against ROS (26). The reduction of GSH is catalyzed by GR in a process that requires NADPH. CsA affects these two GSH dependent antioxidant enzymes and increases the activity of GPx. Therefore, reduced GSH is consumed in this cycle and due to the attenuation of GR activity, it cannot be replaced. Elimination of reduced GSH that plays a central role in the defense against free radicals can be considered as one of the cytotoxic effects of CsA.

Some investigators suggested that CsA-induced oxidative stress is strictly related to biochemical parameters that are responsible for liver toxicity. In the present study, CsA-induced hepatotoxicity was characterized by significant increases in ALT and AST levels and a decrease in urea production in cell supernatants. The liver is the most important site of ammonia metabolism. One major pathway for ammonia detoxification by the liver is urea synthesis so urea production takes place largely within the liver (27). Moreover some investigators have shown a relation between integrin and CsA induced toxicity (11, 28). Alpha2beta1 integrin is an important collagen receptor that not only provides adhesion to collagen, but is also crucial for the regulation of collagen metabolism. Kataoka et al. have shown that CsA induces gingival overgrowth and collagen accumulation. They demonstrated that this effect is due to the inhibition of collagen phagocytosis by ﬁbroblasts, through reduction of α2 integrin expression (29).

In this study, we demonstrated with RT-PCR analysis, that CsA specifically attenuated mRNA expression of α2 integrin subunit in HepG2 cells and mildly reduced the mRNA expression of β1 integrin subunit. These results were confirmed by using real time PCR and were in agreement with some studies on CsA induced gingival overgrowth which was characterized by accumulation of collagenous components in the gingival connective tissue (11, 30). Arora et al. have explained that regulation of extracellular matrix by phagocytosis of collagen fibrils is dependent on the presence of α2β1 integrin, a receptor that mediates initial cellular recognition and binding to collagen fibrils of the ECM (31).

There is currently no evidence regarding the etiology of integrin down-regulation following CsA treatment. It has been shown that integrins are produced in the endoplasmic reticulum with the aid of the protein folding chaperon gp96/grp94 (32). Moreover, CsA has been shown to induce endoplasmic reticulum stress and this effect has been linked to renal fibrosis as a side effect of CsA therapy (33, 34). Further, oxidative stress has been shown to be a side effect of endoplasmic reticulum stress (35). From the mentioned evidence we can hypothesize that the reduction in α2 or β1integrin expression may be a consequence of CsA induced ER stress. Further studies are needed to confirm the validity of this hypothesis.

The effect of ROS on integrin expression should be taken into account. Integrin-mediated signaling has been shown to be affected by oxidation (36). It seems possible that CsA causes diminished α2 and β1 integrin expression by the excess production of ROS. Therefore CsA-induced hepatotoxicity could be due to a reduction in both α2 and β1 integrin subunits expression that may arise either from the direct effect of CsA itself or indirectly through the induction of oxidative stress. Future studies should be conducted, aiming to clarify the exact mechanisms of α2β1 integrin down-regulation and its effect on CsA-induced hepatotoxicity. As the first step towards this endeavor oxidative stress should be induced in hepatocytes by other means and integrin expression should be measured. The result of the aforementioned study will explain whether the reduction of integrin expression following CsA treatment is due to the effect of oxidative stress.
